# Supplementary material for: TORC1 modulation in adipose tissue is required for organismal adaptation to hypoxia in Drosophila
Source: Nat Commun. 2019 Apr 23;10:1878. doi: 10.1038/s41467-019-09643-7 (PMC6478872; doi:10.1038/s41467-019-09643-7)
Supplement: Supplementary file 1 — Supplementary Information [file 41467_2019_9643_MOESM1_ESM.pdf]

**Supplementary Information**

# TORC1 modulation in adipose tissue is required for organismal adaptation to hypoxia in *Drosophila*.

**Lee et al**

a

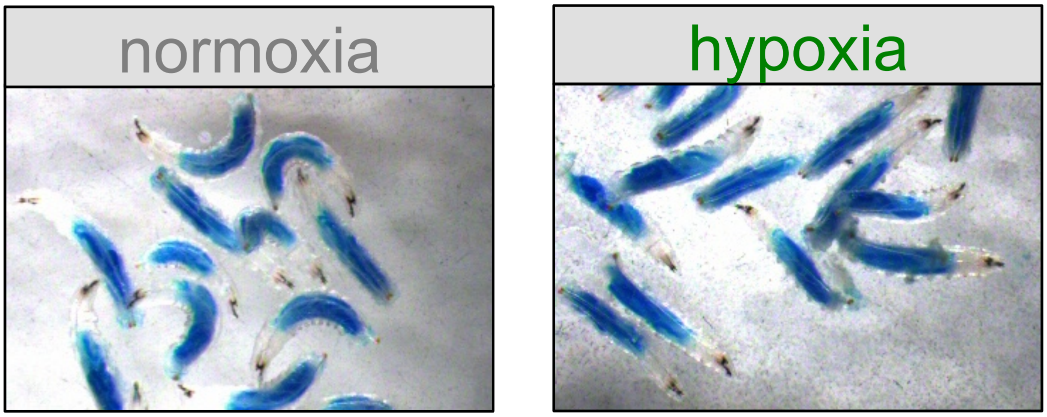


b

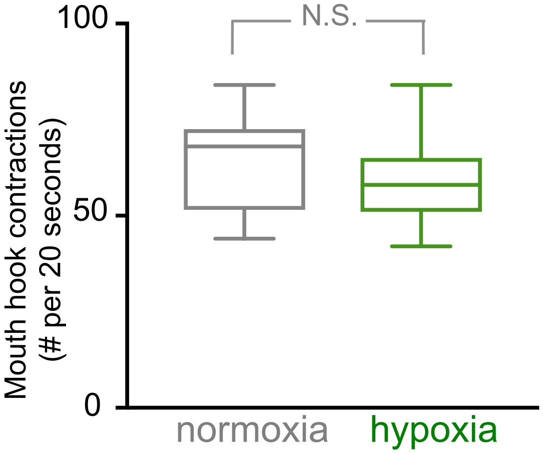


**Supplementary Figure 1. Animals exposed to hypoxia maintain normal food intake. a)** Early third instar larvae were placed into vials containing food mixed with blue food dye. The vials were then either maintained in normoxia or placed into hypoxia for one hour. Larvae were then imaged using a Zeiss Stereomicroscope. The larvae exposed to hypoxia showed similar levels of food intake as larvae maintained in hypoxia. **b)** Early third instar larvae were placed onto a grape juice/agar plate smeared with a small amount of yeast in water. Larvae were then maintained in normoxia or hypoxia for one hour and then the number of mouth hook contractions (number per twenty seconds) was counted using a Zeiss Stereomicroscope. Data are presented as box plots (25%, median and 75% values) with error bars indicating the min and max values. N = 10 animals per experimental condition. There was no significant difference (N.S.) in mouth hook contractions between normoxia and hypoxia exposed larva.

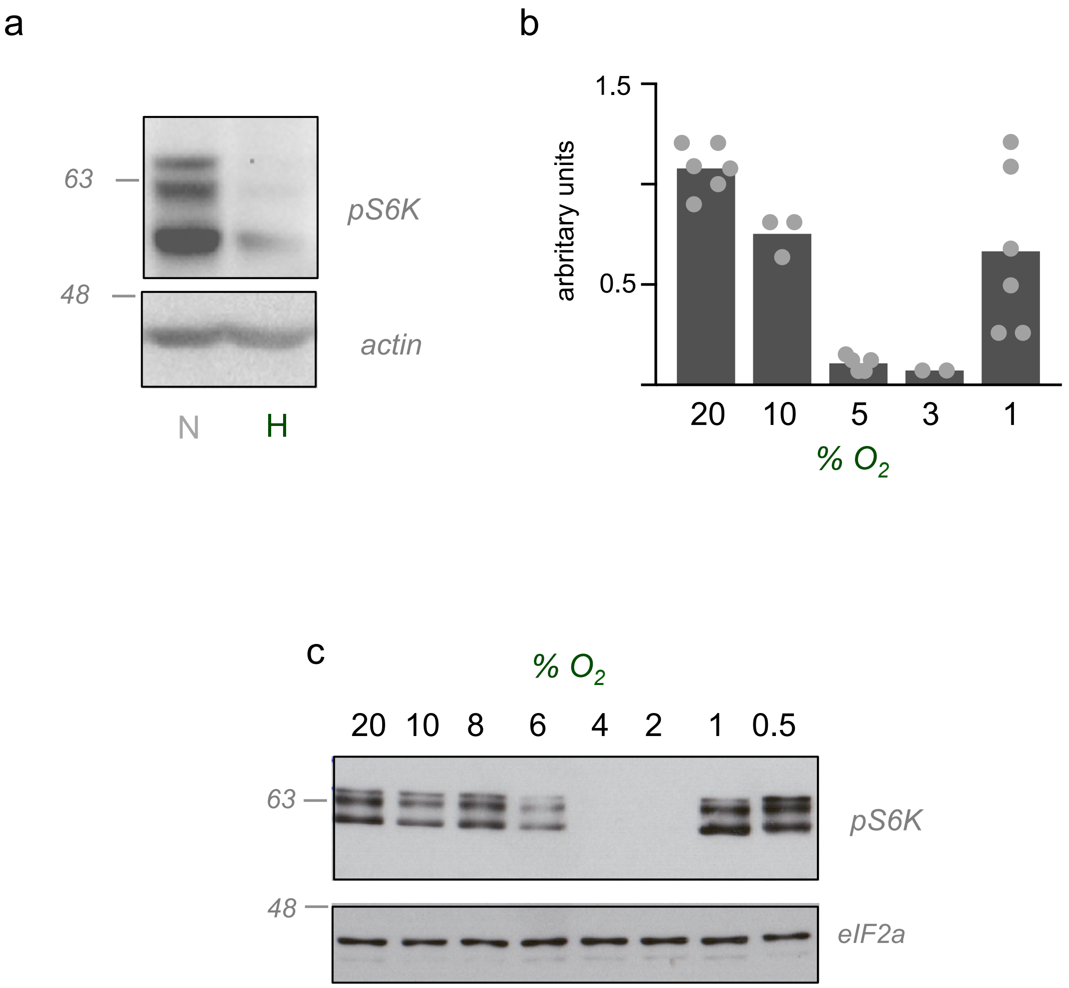


**Supplementary Figure 2. Hypoxia suppresses TORC1 signaling. a)** Early third instar larvae were either maintained in normoxia or transferred from normoxia to hypoxia (5% oxygen) for 40 hours. Larvae were then collected, lysed and processed for SDS-PAGE and western blotting using antibodies to phospho-S6K (pS6K) or actin. **b)** Early third instar larvae were transferred from normoxia to different levels of hypoxia (20-1% oxygen) for 1hr. Larvae were then collected, lysed and processed for SDS-PAGE and western blotting using antibodies to phospho-S6K (pS6K) or total eIF2alpha (eIF2a). The data presented here are the quantified band intensities from the seveal independent experiments. Data represent relative pS6K band intensities (arbitrary units) corrected for eIF2a (loading control) band intensity. Bars indicate mean value and dots indicate the duplicate data points. Quantifications were performed using Image J. Fig 2b in the main paper shows a representative blot. **c)** Early third instar larvae were transferred from normoxia to different levels of hypoxia (20-1% oxygen) for 1hr. Larvae were then collected, lysed and processed for SDS-PAGE and western blotting using antibodies to phospho-S6K (pS6K) or total eIF2alpha (eIF2a).

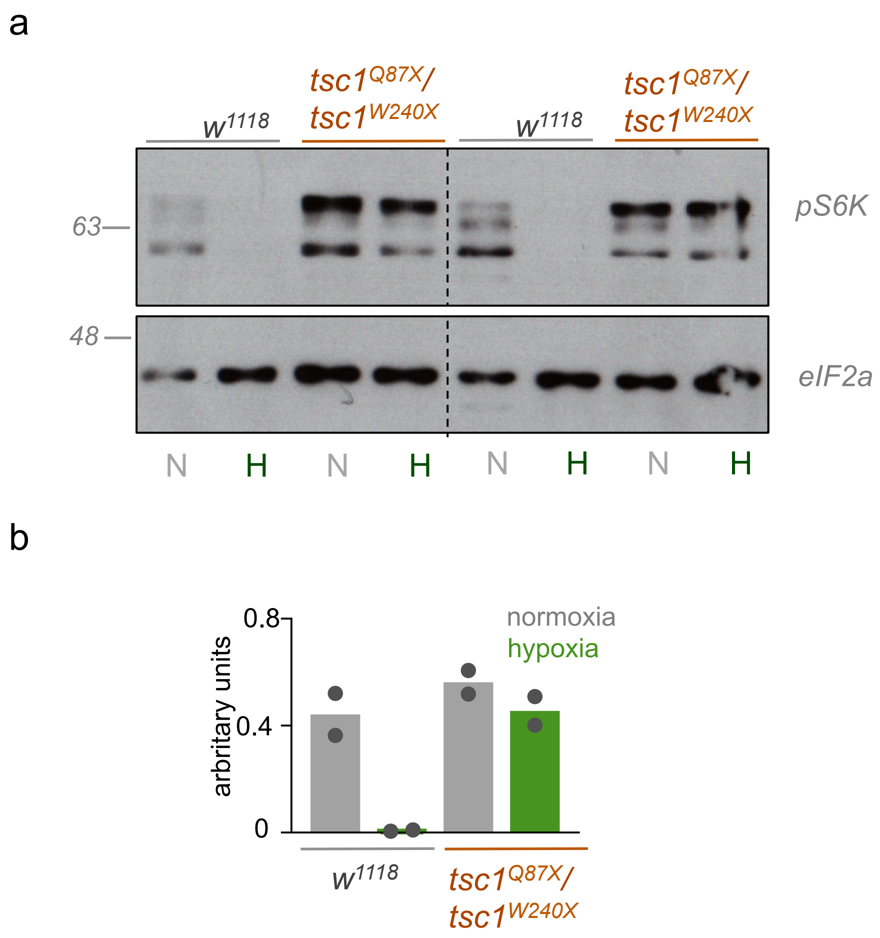


**Supplementary Figure 3. Hypoxia leads to a rapid suppression of TORC1 kinase signaling via activation of TSC1/2 and inhibition of Rheb. a)** Control (*w*$^{1118}$) or tsc1 mutant (*tsc1*$^{Q87X}$/*tsc1*$^{W240X}$) larvae were either maintained in normoxia (N) or transferred from normoxia to hypoxia (5% oxygen, H) for 1hr. Larvae were then collected, lysed and processed for SDS-PAGE and western blotting using antibodies to phospho-S6K (pS6K) or total eIF2alpha (eIF2a). The figure shows result from two independent experiments. **b)** The data presented here are the quantified band intensities from the two independent experiments. Data represent relative pS6K band intensities (arbitrary units) corrected for eIF2a (loading control) band intensity. Bars indicate mean value and dots indicate the duplicate data points. Quantifications were performed using Image J.

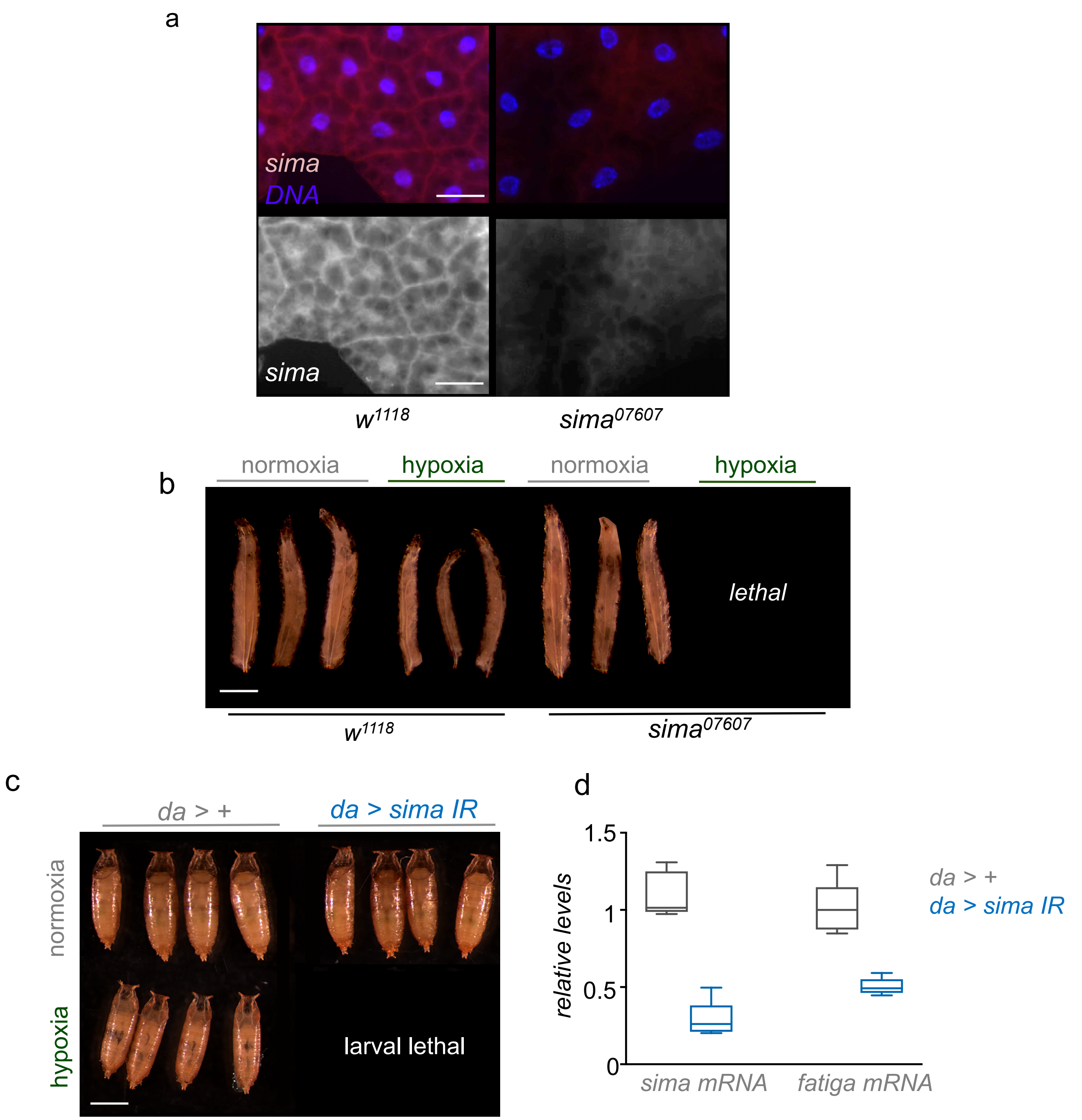


**Supplementary Figure 4.** ***sima*** **mutants and RNAi-mediated knockdown of** ***sima*** **leads to lethality in hypoxia. a)** control ($w^{1118}$) or sima mutant ($sima^{07606}$) larval fat bodies were stained with an antibody to sima. Scale bar = 50mm The nuclear sima staining seen in control samples is absent in sima mutants. **b)** control larvae ($w^{1118}$) or sima mutant larvae ($sima^{07606}$) were maintained in either normoxia or hypoxia (5% oxygen) throughout the larval period. Images of larvae were then taken at 96h after larval hatching. Scale bar =1mm. The sima mutant larvae maintained in hypoxia exhibited 100% lethality. **c)** Control larvae (*da > +)* or larvae ubiquitously expressing an inverted repeat RNAi transgene to *sima* (*da > sima IR*) were maintained in hypoxia throughout the larval period from hatching to pupation. Images of pupae were then captured. Scale bar = 1mm. Knockdown of *sima* lead to complete larval lethality in animals that were maintained in hypoxia. **d)** Control larvae (*da > +)* or larvae ubiquitously expressing an inverted repeat RNAi transgene to *sima* (*da > sima IR*) were lysed at the third instar stage. Total RNA was isolated and levels of *sima* mRNA and *fatiga* mRNA (a sima target gene) measured using qRT-PCR. Data are presented as box plots (25%, median and 75% values) with error bars indicating the min and max values. N=5 independent samples per condition.

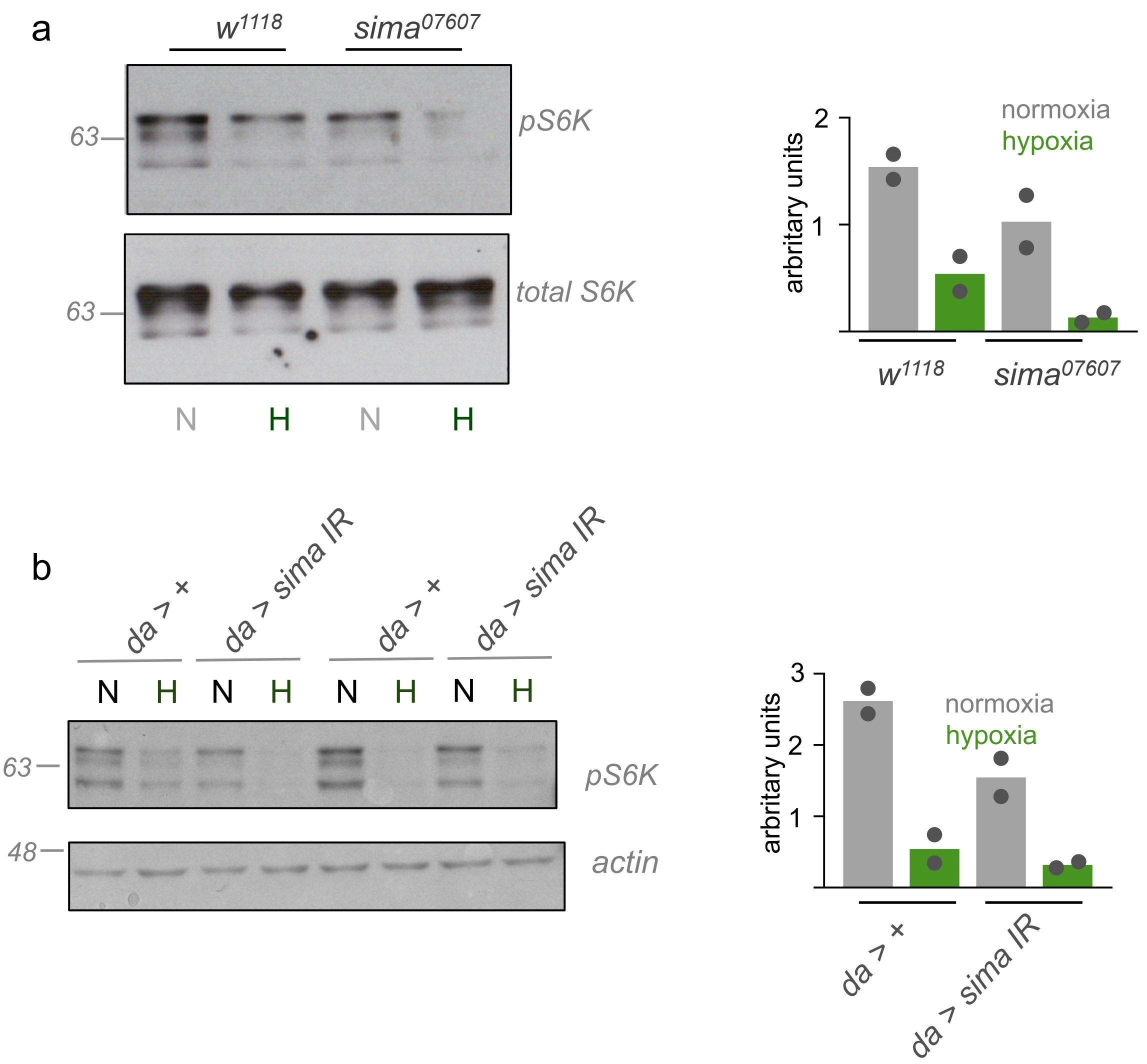


**Supplementary Figure 5. Hypoxia-dependent suppression of TORC1 signaling is independent of *sima*. a)** Left, These data are biological replicates of the data presented in Figure 2e. Control (*$w^{1118}$*) or sima mutant (*$sima^{07606}$*) larvae were either maintained in normoxia (N) or transferred from normoxia to hypoxia (5% oxygen, H) for 1hr. Larvae were then collected, lysed and processed for SDS-PAGE and western blotting using antibodies to phospho-S6K (pS6K) or total S6K. The hypoxia-mediated suppression of TORC1 signaling was still observed in *sima* mutants. Right, the data presented here are the quantified band intensities from the two independent experiments in Fig2e and here. Data represent relative pS6K band intensities (arbitrary units) corrected for total S6K (loading control) band intensity. Bars indicate mean value and dots indicate the duplicate data points. Quantifications were performed using Image J. **b)** Left, Early third instar larvae control larvae (*da > +)* or larvae ubiquitously expressing an inverted repeat RNAi transgene to *sima* (*da > sima IR*) were either maintained in normoxia (N) or transferred from normoxia to hypoxia (5% oxygen, H) for 1hr. Larvae were then collected, lysed and processed for SDS-PAGE and western blotting using antibodies to phospho-S6K (pS6K) or total eIF2alpha (eIF2a). Two independent experimental replicates are shown. Right, the data presented here are the quantified band intensities from the two independent experiments. Data represent relative pS6K band intensities (arbitrary units) corrected for total actin (loading control) band intensity. Bars indicate mean value and dots indicate the duplicate data points. Quantifications were performed using Image J.

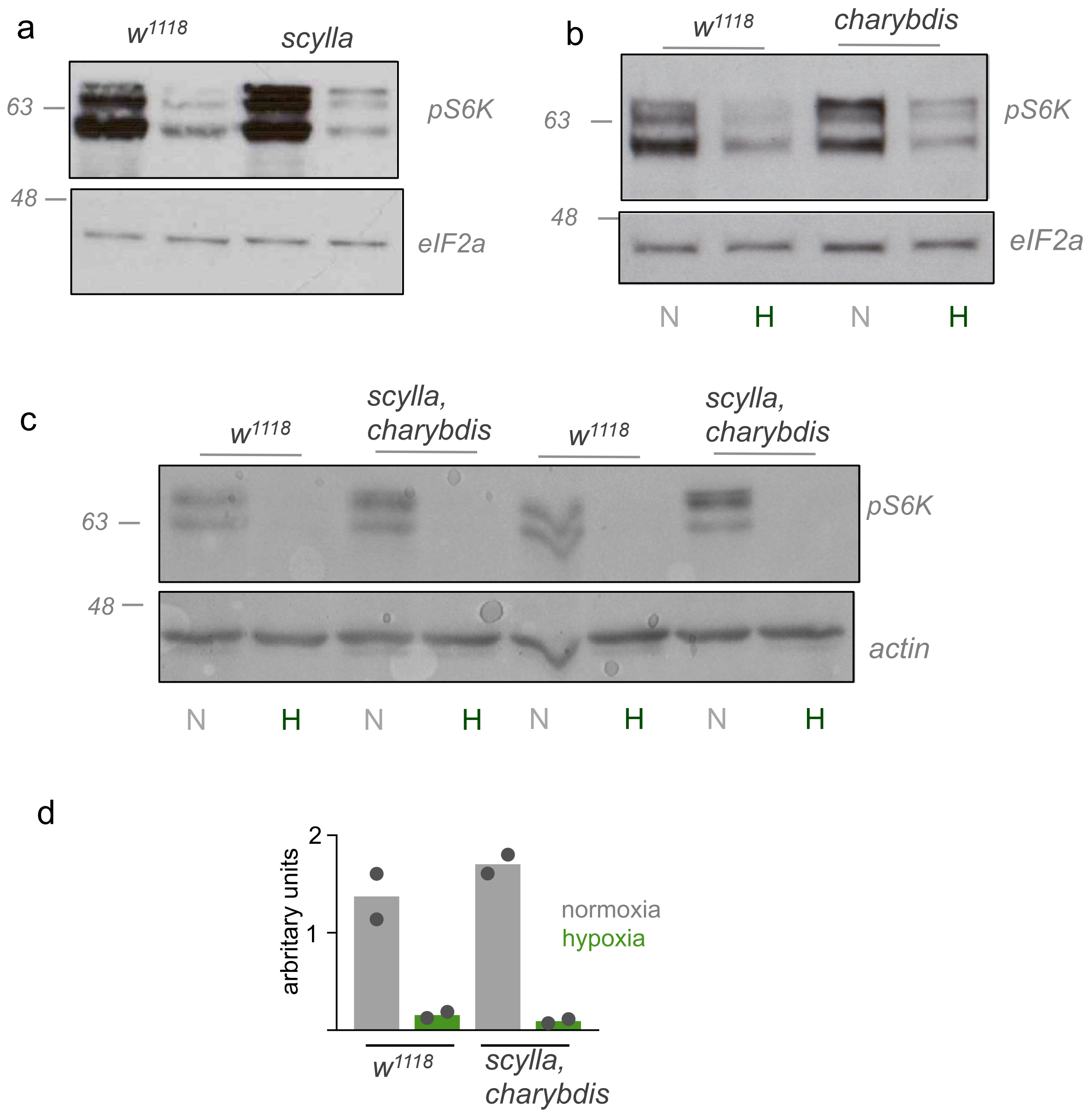


**Supplementary Figure 6. Hypoxia-dependent suppression of TORC1 signaling is independent of *sima* target genes *scylla* and *charybdis*. a)** These data are biological replicates of the data presented in Figure 2f. Early third instar control ($w^{1118}$) or *scylla* mutant (*scylla*) larvae were either maintained in normoxia (N) or transferred from normoxia to hypoxia (5% oxygen, H) for 1hr. Larvae were then collected, lysed and processed for SDS-PAGE and western blotting using antibodies to phospho-S6K (pS6K) or total eIF2alpha (eIF2a). **b)** control ($w^{1118}$) or *charybdis* mutant larvae were either maintained in normoxia (N) or transferred from normoxia to hypoxia (5% oxygen, H) for 1hr. Larvae were then collected, lysed and processed for SDS-PAGE and western blotting using antibodies to phospho-S6K (pS6K) or total eIF2alpha (eIF2a). **c)** control ($w^{1118}$) or *scylla, charybdis* double mutant larvae were either maintained in normoxia (N) or transferred from normoxia to hypoxia (5% oxygen, H) for 1hr. Larvae were then collected, lysed and processed for SDS-PAGE and western blotting using antibodies to phospho-S6K (pS6K) or total eIF2alpha (eIF2a). The figure shows result from two independent experiments (demarcated by dashed lines). **d)** The data presented here are the quantified band intensities from the two independent experiments in **(c).** Data represent relative pS6K band intensities (arbitrary units) corrected for total actin (loading control) band intensity. Bars indicate mean value and dots indicate the duplicate data points. Quantifications were performed using Image J.

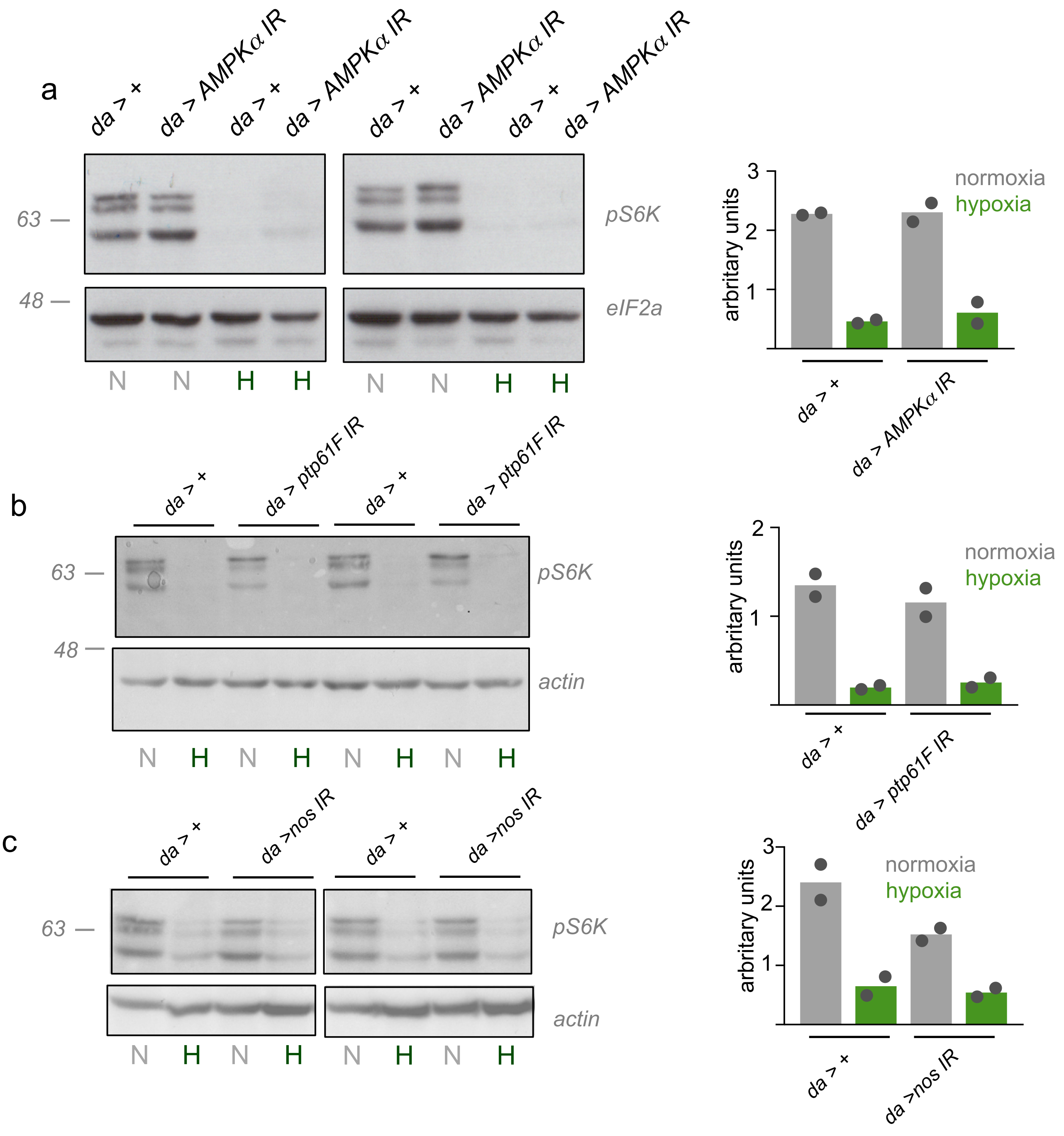


**Supplementary Figure 7. Hypoxia-dependent suppression of TORC1 signaling is independent of AMPK, ptp61F and nitric oxide. a)** Left, Early third instar control larvae (*da* > +) or larvae ubiquitously expressing an RNAi transgene to AMPK*a* (*da >AMPKa IR*) larvae were either maintained in normoxia (N) or transferred from normoxia to hypoxia (5% oxygen, H) for 1hr. Western blots were perfomed on larval lysates using antibodies to phospho-S6K (pS6K) or total eIF2alpha (eIF2a). The figure shows result from two independent biological replicate experiments. Right, the data presented are the quantified band intensities from the two independent experiments. Data represent relative pS6K band intensities (arbitrary units) corrected for eIF2alpha (loading control) band intensity. Bars indicate mean value and dots indicate the duplicate data points. Quantifications were performed using Image J. **b)** Left, Early third instar control larvae (*da* > +) or larvae ubiquitously expressing an RNAi transgene to *ptp61F* (*da >ptp61F IR*) larvae were either maintained in normoxia (N) or transferred from normoxia to hypoxia (5% oxygen, H) for 1hr. Western blots were perfomed on larval lysates using antibodies to phospho-S6K (pS6K) or total eIF2alpha (eIF2a). The figure shows result from two independent biological replicate experiments. Right, the data presented are the quantified band intensities from the two independent experiments. Data represent relative pS6K band intensities (arbitrary units) corrected for actin (loading control) band intensity. Bars indicate mean value and dots indicate the duplicate data points. Quantifications were performed using Image J. **c)** Left, Early third instar control larvae (*da* > +) or larvae ubiquitously expressing an inverted repeat RNAi transgene to *nos* (*da >nos IR*) larvae were either maintained in normoxia (N) or transferred from normoxia to hypoxia (5% oxygen, H) for 1hr. Western blots were perfomed on larval lysates using antibodies to phospho-S6K (pS6K) or total eIF2alpha (eIF2a). The figure shows result from two independent biological replicate experiments. Right, the data presented are the quantified band intensities from the two independent experiments. Data represent relative pS6K band intensities (arbitrary units) corrected for actin (loading control) band intensity. Bars indicate mean value and dots indicate the duplicate data points. Quantifications were performed using Image J.

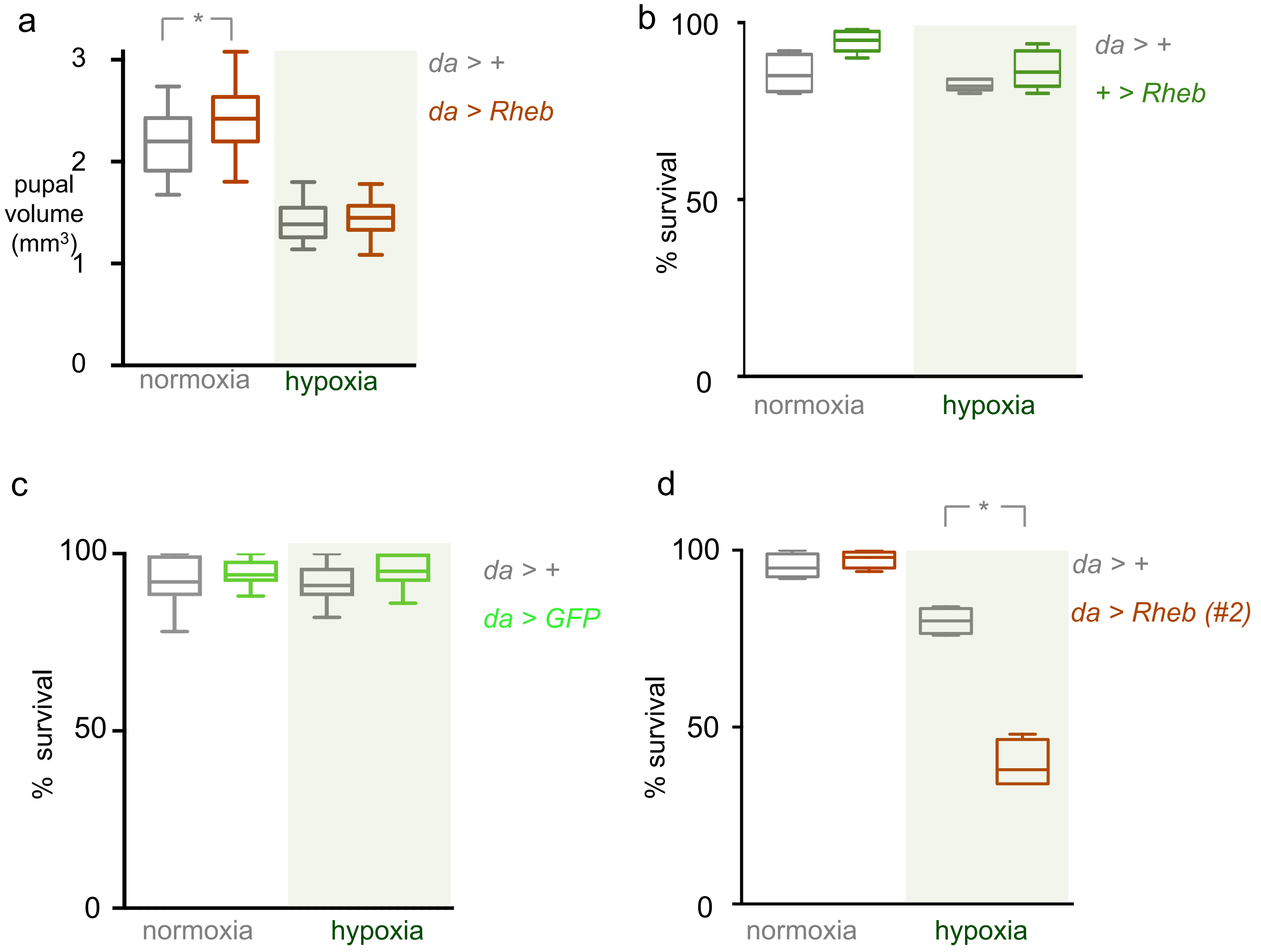


**Supplementary Figure 8. Suppression of TORC1 is required for adaptation to hypoxia**. **a)** Control (*da* > +) or Rheb overexpressing (*da* > *Rheb*) animals were maintained in either normoxia or hypoxia (5% oxygen) throughout the larval period. Pupal volume was calculated for each experimental condition. Data are presented as box plots (25%, median and 75% values) with error bars indicating the min and max values. N >100 pupae per condition. * = $p<0.05$, Students t-test. **b)** Larvae carrying either the *da-Gal4 (da>+)* or the *UAS-Rheb (+>Rheb)* transgene were maintained in either normoxia or hypoxia (5% oxygen) throughout the larval period and then were returned to normoxia at the beginning of the pupal stage. The percentage of animals that eclosed as viable adults was measured. Data are presented as box plots (25%, median and 75% values) with error bars indicating the min and max values. N= 4 groups of animals (50 animals per group) per experimental condition. **c)** Control (*da* > +) larvae or larvae overexpressing UAS-GFP (*da>GFP*) were maintained in normoxia or hypoxia (5% oxygen) throughout the larval period and then were returned to normoxia at the beginning of the pupal stage. The percentage of animals that survived to adults was then measured. Data are presented as box plots (25%, median and 75% values) with error bars indicating the min and max values. N= 12 independent groups of animals (50 animals per group) per experimental condition. Expression of GFP had no effect on survival. **d)** Control (*da* > +) larvae or larvae overexpressing a second independent UAS-Rheb transgene (*da >Rheb#2*) were maintained in either normoxia or hypoxia (5% oxygen) throughout the larval period and then were returned to normoxia at the beginning of the pupal stage. The percentage of animals that eclosed as viable adults was then measured. Animals expressing Rheb and exposed to hypoxia as larvae showed a significant decrease in adult survival. Data are presented as box plots (25%, median and 75% values) with error bars indicating the min and max values. N= 4 independent groups of animals (50 animals per group) per experimental condition. * = $p<0.05$, Students t-test.

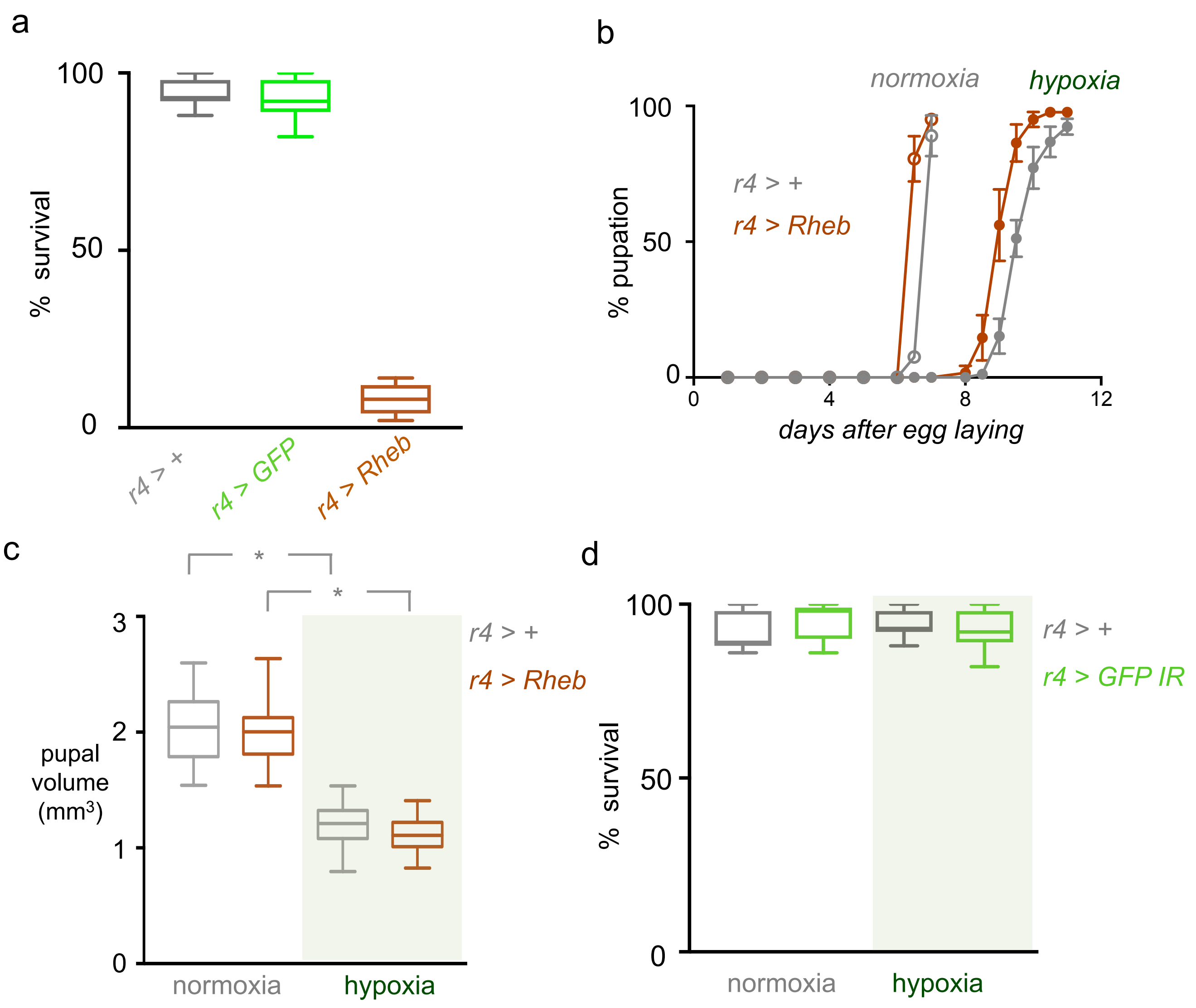


**Supplementary Figure 9. Fat-specific suppression of TORC1 is required for adaptation to hypoxia**. **a)** Control larvae (*r4 > +*), larvae overexpressing UAS-GFP in the fat body (*r4 >GFP*), or larvae overexpressing Rheb (*r4>Rheb*) were maintained in hypoxia (5% oxygen) throughout the larval period and then were returned to normoxia at the beginning of the pupal stage. The percentage of animals that survived to adults was then measured. Data are presented as box plots (25%, median and 75% values) with error bars indicating the min and max values. N= 7 groups of animals (50 animals per group) per experimental condition. **b)** Control larvae (*r4 > +*) or larvae overexpressing Rheb in the fat body (*r4 >Rheb*) were maintained in either normoxia or hypoxia (5% oxygen) throughout the larval period. The rate of larval development was measured by calculating the percentage of animals that progressed to the pupal stage over time. Maintaining TORC1 signalling in the larval fat body did not reverse the hypoxia-mediated delay in larval development. Data points represent mean +/- SEM, N=4 groups of animals per experimental condition (50 animals per group) **c)** Control larvae (*r4 > +*) or larvae overexpressing Rheb in the fat body (*r4 >Rheb*) were maintained in either normoxia or hypoxia (5% oxygen) throughout the larval period. Pupal volumes were then measured for each experimental condition. Data are presented as box plots (25%, median and 75% values) with error bars indicating the min and max values. N >100 pupae per condition. * = $p<0.05$, Students t-test. **d)** Control larvae (*r4 > +)* or larvae expressing an inverted repeat RNAi transgene to *GFP* (*r4 > GFP IR*) were maintained in hypoxia throughout the larval period from hatching to pupation. Animals were then returned to normoxia and the percentage of animals surviving to the adult stage counted. Data are presented as box plots (25%, median and 75% values) with error bars indicating the min and max values. N= 8 groups of animals (50 animals per group) per experimental condition. Expression of GFP IR had no effect on survival.

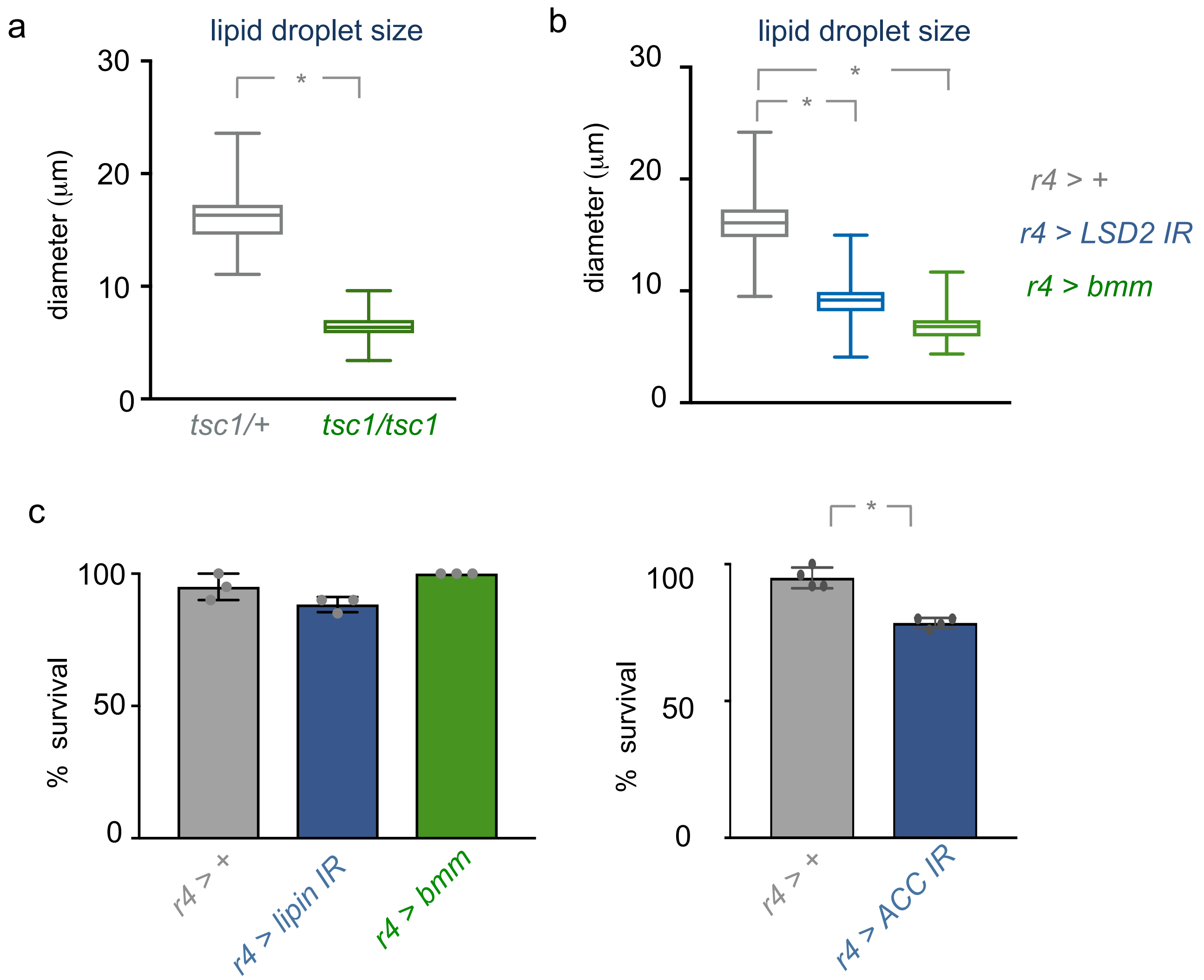


**Supplementary Figure 10. Lipid droplet mobilization is required for hypoxia adaptation. a) (related to Fig 7)**The MARCM system was used to generate GFP-marked *tsc1*$^{W240X}$ mutant cell clones in the fat body. Hatched larvae were then either maintained in normoxia or transferred to hypoxia. At the third instar stage, larval fat bodies were fixed, dissected and mounted on coverslips. Fat bodies were then imaged using DIC microscopy to visualize lipid droplets. Lipid droplet diameters from *tsc1* mutant cells (GFP-marked) and surrounding non-GFP-marked cells (*tsc1*/+ or +/+) were measured. Data here are presented as box plots (25%, median and 75% values) with error bars indicating the min and max values. N>30 per condition. * = p<0.05, Students t-test. Representative images are shown in Fig 7. **b) (related to Fig 8).** Control larvae (*r4 > +)* , larvae expressing an inverted repeat RNAi transgene to Lsd2 (*r4 > Lsd2 IR*), or larvae expressing *brummer* (*r4>bmm*) were transferred to hypoxia at 72hrs. After 48 hours of hypoxia, fat bodies were dissected and stained with Nile Red. Lipid droplet diameters were then measured. N>70 per condition. Data here are presented as box plots (25%, median and 75% values) with error bars indicating the min and max values. * = p<0.05, Students t-test. Representative images are shown in Fig 8a. **c) (related to Fig 8). Left,** Control larvae (*r4 > +)* or larvae expressing either an inverted repeat RNAi transgene to *lipin* (*r4>lipin IR*) or overexpressing *brummer* (*r4 >bmm*) were maintained in normoxia and the percentage of animals surviving to the adult stage counted. Data are presented as box plots (25%, median and 75% values) with error bars indicating the min and max values. N= 3 groups of animals (50 animals per group) per experimental condition. Right panel, Control larvae (*r4 > +)* or larvae expressing either an inverted repeat RNAi transgene to *ACC* (*r4>ACC IR*) were maintained in normoxia and the percentage of animals surviving to the adult stage counted. Data are presented as box plots (25%, median and 75% values) with error bars indicating the min and max values. N= 4 groups of animals (50 animals per group) per experimental condition.
